# Genomic Signatures of Selection Reveal Breed-Specific and Shared Adaptive Regions in South African Beef Cattle

**DOI:** 10.3390/ani16111645

**Published:** 2026-05-28

**Authors:** Mamokoma Cathrine Modiba, Aletta Matshidiso Magoro, Peter Ayodeji Idowu, Khathutshelo Agree Nephawe, Jabulani Nkululeko Ngcobo, Takalani Judas Mpofu, Bohani Mtileni

**Affiliations:** Department of Animal Sciences, Tshwane University of Technology, Pretoria 0001, South Africa; alettamgr@gmail.com (A.M.M.); ayodejiidowuolu@gmail.com (P.A.I.); ngcobojn@tut.ac.za (J.N.N.); mpofutj@tut.ac.za (T.J.M.); mtilenib@tut.ac.za (B.M.)

**Keywords:** candidate genes, fixation index, integrated haplotype score, traits, adaptive divergence

## Abstract

In genetics and evolutionary biology, selection signatures refer to distinct genomic patterns that reflect the action of natural and artificial selection on populations over time. Detecting such signatures provides critical insights into adaptive evolution and breed differentiation, especially in livestock populations subjected to diverse production environments and breeding objectives. In this study, a total of 96 samples were collected from four different cattle breeds, namely, South African indigenous Nguni (n = 28), Bonsmara (n = 21), Angus (n = 22), and Simmental (n = 25). The samples were genotyped using the Illumina Bovine SNP 150K BeadChip and subjected to quality control. Selection signatures were identified using the integrated haplotype score (iHS) method and the fixation index (Fst) method to assess the genetic differences between breeds. The complementary application of within-population and cross-population approaches enabled the detection of both recent and divergent selective pressures. A total of twelve regions were found to be under selection, with *Bos taurus* autosome (BTA) 12 being common between Nguni and Bonsmara. Gene annotation analyses identified several genes, including *FAM110B*, *CDK8*, and *FLT1* in Bonsmara cattle, whereas Nguni cattle indicated potential genes such as *CRB1*, *PLA2G4A*, and *VASH2*, with *CDK8* common between Bonsmara and Nguni on BTA 12. Cross-population analyses further identified *PLCXD3*, *FAM149B1*, and *GRIK2* as candidate genes differentiating Bonsmara from Nguni cattle, and *TSPAN9* distinguishing Simmental from Angus cattle. These results indicated breed-specific adaptive divergence. The study revealed genomic regions that are under selection in South African Nguni, Bonsmara, and Simmental cattle, with less information for Angus cattle breeds. Several candidate genes were found to be associated with reproductive traits (such as sperm count and inseminations per conception), disease resistance (such as bovine respiratory disease), and calving ease. This study identifies breed-specific and shared genomic regions under selection across diverse cattle breeds, providing novel insights into the genetic basis of adaptation and production-related traits. These findings explain the potential application of selection signature analyses in genomic-assisted breeding programmes aimed at improving productivity, resilience, and sustainability of cattle populations.

## Simple Summary

South African beef cattle represent a genetically diverse system shaped by both indigenous adaptation and structured breeding programmes. The genomic structure of livestock has undergone significant transformations since its domestication. These changes have been driven by a combination of natural selection, human-mediated breeding practices, and adaptation to diverse agro-ecological environments, resulting in distinct breed-specific genomic architectures. This study investigated genomic signatures of recent positive selection and genomic differentiation among four South African beef cattle breeds: Bonsmara, Nguni, Simmental, and Angus using high-density SNP data. The discovery of selection signatures provides new perspectives and identifies potential genes linked to reproductive characteristics in native South African cattle breeds. These results provide insightful information that can guide future research and breeding and selection initiatives targeted at enhancing reproductive performance and adaptation.

## 1. Introduction

Selection signatures are distinct patterns in the genome that develop as a result of natural or artificial selection processes [1]. These genetic footprints reflect past and continuing selective pressures on populations and can be identified by differences in allele frequencies, haplotype structure, or levels of population divergence [2]. Innovations in high-throughput genotyping technologies and the development of robust statistical approaches have significantly improved the ability to discover and analyse selection signatures in domestic animal species [3,4,5]. The genomic structure of livestock has undergone significant transformations since their domestication [3]. The Bonsmara is a composite South African beef cattle breed developed in the early 1940s through a structured breeding programme designed to combine adaptation and productivity. The breed comprises a 5/8 to 3/8 genetic contribution from the indigenous Afrikaner (Sanga cattle; *Bos taurus africanus*) and British beef breeds, primarily Shorthorn and Hereford (*Bos taurus*) [6]. The Nguni breed, an ecotype of Sanga cattle (*Bos taurus africanus*), is believed to have originated from ancient crossbreeding events between Zebu cattle (*Bos indicus*) and humpless Hamitic Longhorn and Shorthorn cattle in East and Central Africa, resulting in a population well adapted to diverse African environments [7]. The Simmental breed, which originated from crosses between German and Swiss cattle, remains one of the most widely utilised breeds in structured breeding programmes due to its adaptability and production performance [8]. Similarly, the Scottish Angus breed has been present in South Africa for over a century and is formally recorded in the South African Stud Book, reflecting its long-standing contribution to the national beef cattle industry [9]. Livestock domestication has resulted in many changes in morphology, behaviour, and productivity traits, particularly during the early stages of selection [3]. These changes were driven by controlled breeding, management, and feeding practices imposed by humans, as well as by the progressive isolation of domestic populations from their wild ancestors [5]. South African beef cattle represent a genetically diverse system shaped by both indigenous adaptation and structured breeding programmes.

This diversity reflects the widespread use of crossbreeding to enhance productivity [4]. However, this practice has raised concerns regarding the erosion of indigenous genetic resources, especially in locally adapted breeds such as Nguni cattle [10,11]. Therefore, understanding the genomic consequences of domestication and crossbreeding is essential for sustainable beef production systems. Several genome-wide studies have identified selection signatures associated with economically important traits in cattle, including growth, reproduction, disease resistance, and environmental adaptation. Although individual breeds have been previously studied, comparative genome-wide selection signature analyses that include both indigenous (Nguni), composite breeds (Bonsmara), and commercial (Angus and Simmental) breeds using a consistent high-density SNP platform are, in the South African context, relatively scarce. This represents a limitation that constrains our understanding of breed-specific and shared genetic adaptations in the South African beef cattle system. Makina [4] conducted one of the first comprehensive studies of selection signatures within South African cattle breeds, particularly in Afrikaner, Nguni, Drakensberg, Bonsmara, Angus, and Holstein, using the Illumina BovineSNP50 Bead Chip. The analysis used haplotype fixation and Fst methodologies to pinpoint selective sweeps and divergent genomic regions, thereby identifying potential genes associated with adaptation, production, and reproductive traits, but using lower-density SNPs. A more recent study identified selection signatures associated with adaptation and production traits in Nguni and Bonsmara populations using a 150K SNP panel. However, the study focused only on South African indigenous breeds [12]. Although Gomo [13] used runs of homozygosity and Fst to detect signatures of adaptation in Drakensberg, Nguni, and Tuli, comparative genome selection scans using a consistent high-density SNP platform and multiple complementary selection methods across both indigenous and commercial breeds remain limited and motivate the present study. Local composites have unique morphological characteristics that distinguish them from other cattle breeds; they also have underlying traits such as disease resistance, climatic stress resistance, and productivity traits [14], while commercial breeds’ selection signatures are more often related to production efficiency, carcass, and reproductive traits [12].

Selection signatures in South African beef cattle can be studied using different methods frequently used in population genetics to pinpoint genomic areas that undergo selection. These include integrated haplotype score (iHS) and fixation index (Fst) [15]. The iHS identifies selection within a breed by detecting SNPs at which one allele is associated with one or a few long haplotypes. This approach has also been described as an efficient method for detecting genomic areas with segregated mutations for complex traits under selection [16]. While the Fst approach is most effective when there are significant allele frequency differences between pairs of breeds, a population that has undergone selection will differ from other populations [16].

Therefore, this study aims to identify and compare genome-wide patterns of positive and divergent selection in South African beef cattle breeds using iHS and Fst analyses, using high-density SNP genotyping across Nguni, Bonsmara, Angus, and Simmental cattle.

## 2. Materials and Methods

### 2.1. Ethics

This study was approved by the Animal Research Ethics Committee of the Agricultural Research Council (APIEC17/17). All experimental procedures were conducted in accordance with the ARRIVE guidelines for reporting animal research.

### 2.2. Populations and Sample Size

Ninety-six semen samples from Bonsmara, n = 21; Nguni, n = 28; Simmental, n = 25; and Angus, n = 22, were randomly collected from four provinces: Mpumalanga (25.4294° S, 29.3306° E), Northwest Province (25.6318° S, 27.7829° E), Gauteng (26.2708° S, 28.1123° E), and Limpopo (23.0353° S, 29.6583° E) in South Africa. Sampling was performed to minimise close relatedness among individuals within each breed. Semen samples were subsequently cryopreserved and stored at the Agricultural Research Council (Animal Production Germplasm Conservation and Reproductive Biotechnologies) in Irene, South Africa.

### 2.3. DNA Extraction, Genotyping, and Quality Control

DNA was extracted from semen samples using a NucleoMag^®^ pathogen (MACHEREY-NAGEL, Duren, Germany) extraction kit in accordance with the manufacturer’s instructions. DNA was quantified using Qubit^®^ 2.0 and subsequently genotyped with the Illumina BovineSNP 150K BeadChip (illumina, San Diego, CA, USA) according to the manufacturer’s instructions. SNP-generated data were then visualised and interpreted using Genome Studio 2.0 software. Plink v1.07 [17] software was used to filter the dataset based on the following criteria: excluding SNPs with call rate frequencies of less than 90% (due to low-frequency SNPs being missing in many individuals, which can reduce the reliability of analyses). Population structure analysis was conducted using the LEA [18] package version 3.18.0 in R [19]. The most probable number of ancestral populations (K) was estimated by running sparse non-negative matrix factorisation (sNMF) models with cross-entropy criterion, as implemented in LEA. The StructuRly database was then utilised to classify the remaining individuals based on population using triangle plots.

### 2.4. Identifying Signatures of Selection

To assess within-population selection signals, the iHS approach was utilised to identify regions of selection within cattle populations. Then, to evaluate genetic differences among populations, Fst was used in a pairwise approach to determine the regions under positive selection that are shared across the four cattle populations.

### 2.5. The iHS Analysis

The integrated haplotype score (iHS) approach was applied to compare extended haplotype homozygosity (EHH) between derived and ancestral alleles within each cattle population [20]. Signatures were found across the four cattle populations, and following quality control, breed-specific binary files were generated using PLINK v1.9. The binary files were subsequently converted to Variant Call Format (VCF) and sorted by base-pair position using BCFtools [21], and phasing of the genotype data was performed using BEAGLE v5 [22]. The iHS statistics were calculated in R v4.2.2 using the *rehh* package [23]. Candidate regions were initially identified using a minor allele frequency (MAF) threshold of <0.05, while genomic regions under strong selection were defined using a stringent significance threshold of *p* < 0.0001. The results for each population were visualised as Manhattan plots in R v4.2.2, and significant genomic regions exceeding the threshold of −log10(|iHS|) > 4 were extracted and exported as comma-separated value (.csv) files for downstream analyses.

### 2.6. The Fst Analysis

Fst [24] was calculated to assess genetic differentiation between populations, and regions with Fst values greater than 4 were considered as candidate loci potentially under selection [25]. The Fst computation was performed in PLINK v1.9 using the Fst command, and Manhattan plots were generated for visualisation using the qqman [26] package in R.

### 2.7. Gene Functional Annotation

Gene annotation was performed on genomic regions identified as exhibiting positive signatures of selection, as determined by both iHS and Fst analyses. The cattle gene assembly ARS-UCD1.2 was used to annotate the genes using BioMart, in Ensembl database, and ShinyGO v.0.77 to identify gene functions, and Draw.OI was used to generate the Venn diagram for all overlapping genes and their functions.

## 3. Results

**Figure 1:**
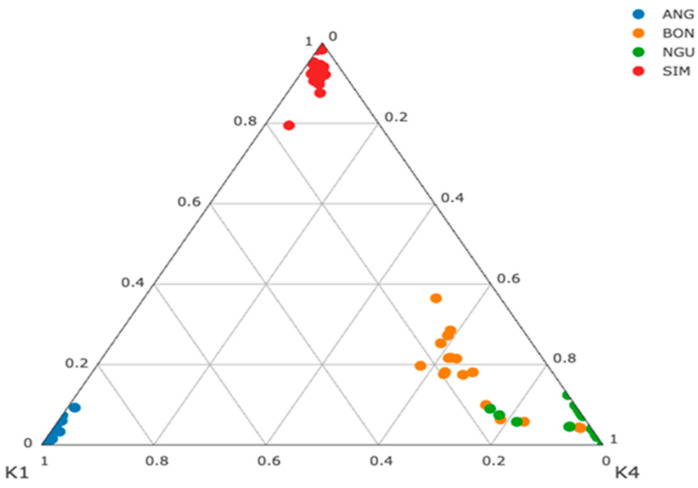
Population structure of individual animals inferred using ADMIXTURE analysis at K = 4, with colours indicating genetic ancestry corresponding to Bonsmara (BON), Nguni (NGU), Simmental (SIM), and Angus (ANG) breeds.

Figure 1 indicates that population structure analysis revealing four clear genetic clusters among cattle breeds. The Angus (ANG: blue) individuals clustered predominantly near K1, while the Bonsmara (BON: orange) individuals were distributed between K1 and K4, with individuals closer to K4, reflecting moderate admixture but primarily alignment with cluster 4. The Nguni (NGU: green) individuals clustered closely with Bonsmara near K4, suggesting a high proportion of their genome is derived from cluster 4 and highlighting their shared genetic background with Bonsmara, and Simmental (SIM: red) individuals showed a high proportion of ancestry from cluster 0, indicating genetic homogeneity and limited admixture relative to the Bonsmara and Nguni.

### 3.1. Genome-Wide Identification of Recent Positive Selection Using iHS

**Figure 2:**
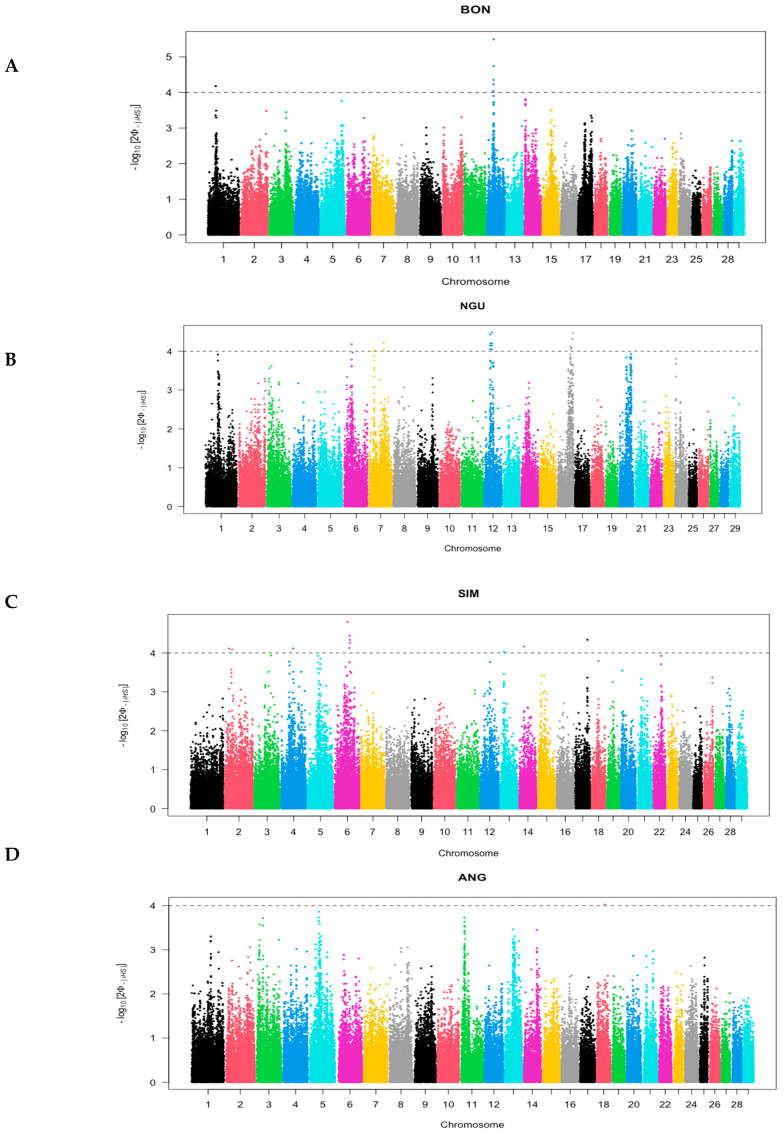
Manhattan plot iHS results for (**A**) Bonsmara (BON), (**B**) Nguni (NGU), (**C**) Simmental (SIM), and (**D**) Angus (ANG) cattle across all 29 chromosomes. The *x*-axis represents chromosome number, and the *y*-axis represents (−log10, *p*-value) for each SNP. Peaks above y = 4 indicate SNPs with *p* < 0.001, representing highly significant SNPs. Each colour represents a different chromosome for visual distinction.

The iHS results were visualised using Manhattan plots, which identified selection signatures within each breed across the four beef cattle breeds: Bonsmara, Nguni, Simmental, and Angus. Genomic regions that were above the significance threshold (*p* < 0.001) were under recent positive selection. Bonsmara (Figure 2A) revealed significant selection signals on BTA 12. Nguni (Figure 2B) revealed significant regions on BTA 6, 7, 12, and 16. Simmental (Figure 2C) had several significant regions on BTA 2, 4, 6, 13, 14, and 17. In contrast, Angus (Figure 2D) had a single significant region on BTA 18, with weaker selection signals. In total, twelve important genomic regions were identified across the four breeds studied. Notably, BTA 12 revealed a common selection signal in both the Nguni and Bonsmara breeds.

### 3.2. Genome-Wide Patterns of Genetic Differentiation Detected by Fst

**Figure 3:**
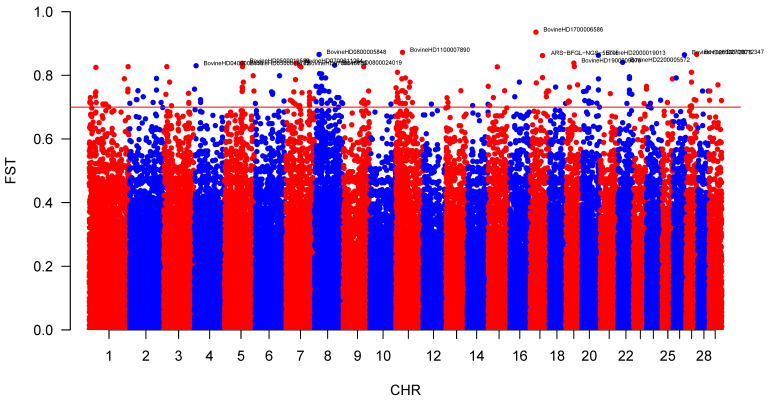
Manhattan plot for Fst between Bonsmara and Nguni cattle, horizontal red line indicates the threshold for high genetic differentiation, and the labeled SNPs are those exceeding this threshold, suggesting potential regions under divergent selection.

**Figure 4:**
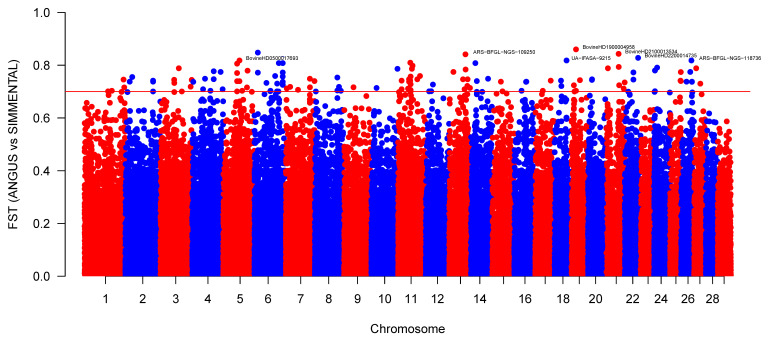
Manhattan plot for Fst between Angus and Simmental cattle, horizontal red line indicates the threshold for high genetic differentiation, and the labeled SNPs are those exceeding this threshold, suggesting potential regions under divergent selection.

Genome-wide population differentiation between the cattle breeds was assessed using fixation index (Fst) Manhattan plots. A horizontal line was employed in each comparison to identify outlier thresholds, thereby revealing SNPs demonstrating a significant level of genetic differentiation. In Figure 3, the threshold for the Bonsmara vs. Nguni comparison was established at approximately Fst = 0.4, whereas in Figure 4, a more stringent cut-off was observed in the Angus vs. Simmental comparison (Fst = 0.7) which reflected a clear overall divergence between the breeds. SNPs exceeding the threshold were subsequently classified as putative candidates under divergent selection. The identified outlier loci were then prioritised for further analysis.

### 3.3. Gene Annotation

Gene annotation analyses were conducted, and the results are presented in Table 1. For Bonsmara cattle, two candidate genes, CDK8 and FLT1, located on BTA 12, were identified. Nguni cattle displayed candidate genes including CRB1, PLAG2GA, UBL3, VASH2, and ENSBTAG00000019340, with CDK8 shared between the Bonsmara and Nguni breeds on BTA 12. No candidate genes were detected on BTA 18 for Angus cattle. For Simmental cattle, several significant genes were identified, including FAM110B, RBMS1, SRD5A3, TBX5, and TMEM165, with FAM110B notably associated with quantitative trait loci (QTLs) related to carcass weight (CW) and body conformation (BC). Population comparisons using the Fst method are summarised in Table 2. For Bonsmara vs. Nguni, candidate genes included PLCXD3, FAM149B1, TCAIM, AFG1L, FOXO3, KCNQ3, PAPPA, PLXDC2, ZNF704, and GRIK2. For the Simmental vs. Angus comparison, candidate genes identified included MYOID, FAM172A, MIPOL1, CHMP4B, and KCNK13.

### 3.4. Gene Functions

**Figure 5:**
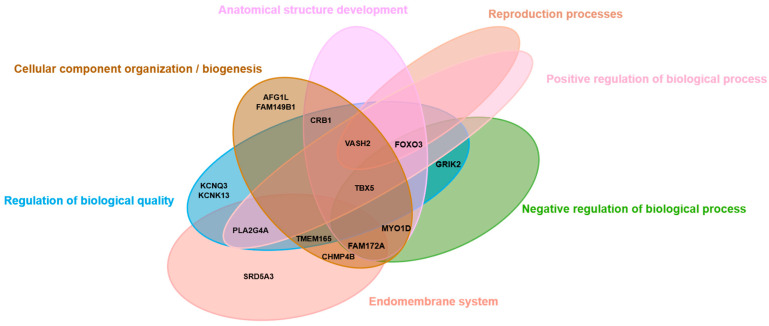
Overlap of genes involved in key biological processes related to growth, reproduction, and cellular organisation.

The biological functions of the genes identified in Figure 5 in this study reveal their involvement in multiple biological processes relevant to cellular function, development, and reproduction. Genes associated with cellular component organisation or biogenesis included AFG1L, CHMP4B, MYO1D, FAM149B1, FAM172A, TMEM165, VASH2, CRB1, and TBX5, highlighting their potential roles in maintaining cellular architecture and organelle formation. Several genes, such as FOXO3, GRIK2, MYO1D, FAM172A, and TBX5, were associated with the negative regulation of biological processes, suggesting a role in controlling cellular homeostasis. Genes like TMEM165, CRB1, TBX5, PLA2G4A, FOXO3, KCNQ3, GRIK2, and KCNK13 were involved in the regulation of biological quality, reflecting their potential contribution to maintaining physiological integrity. Reproductive processes were associated with VASH2 and FOXO3, indicating their importance in fertility and reproductive performance. Additionally, genes such as FOXO3, FAM172A, VASH2, CRB1, and TBX5 were linked to anatomical structure development, emphasising roles in growth and morphogenesis. Positive regulation of biological processes was predominantly influenced by VASH2, TBX5, PLA2G4A, and FOXO3, while genes associated with the endomembrane system, including CHMP4B, FAM172A, TMEM165, PLA2G4A, and SRD5A3, suggest their involvement in intracellular trafficking and membrane dynamics. Collectively, these genes demonstrate overlapping functions across multiple biological processes, highlighting their pleiotropic roles in growth, reproduction, cellular organisation, and homeostatic regulation.

## 4. Discussion

The present study investigated population structure, recent positive selection, and genomic differentiation among four South African beef cattle breeds: Bonsmara, Nguni, Simmental, and Angus using high-density SNP data. The population structure analysis revealed clear genetic clustering at K = 4, consistent with the four breeds selected for the study. The distinctive genetic differences between Bonsmara and Nguni, as well as Angus and Simmental, indicate their different origins and breeding histories. Moreover, Bonsmara and Nguni revealed evidence of genetic admixture, which is consistent with the composite nature of the Bonsmara breed and its historical evolution involving both *taurine* and indigenous African cattle. This was supported by a study using whole-genome single-nucleotide polymorphism (SNP) analyses of eight South African beef breeds, including Bonsmara, Nguni, Beefmaster, Boran, Charolais, Hereford, Drakensberg, and Tuli, which revealed three biological groups: indigenous, composite, and exotic. The study further showed a clear separation between the Bonsmara and Nguni breeds, while the composite breeds displayed evidence of admixture, with phylogenetic trees revealing that Bonsmara, Tuli, Nguni, and Boran all share common ancestry [27,28]. Furthermore, there was a distinct difference between Angus and Simmental, supporting their relatively strict breeding systems and strong artificial selection for specific production traits. Overall, these clustering patterns are consistent with known breed histories and past population genetic research on beef cattle.

### 4.1. Within-Population Selection Signals and Shared Genomic Regions

The iHS analysis revealed several genomic regions under recent positive selection within individual breeds, highlighting breed-specific adaptation and production-related pressures. In Bonsmara cattle, a strong selection signal was detected on BTA 12, while Nguni cattle exhibited multiple significant regions on BTA 6, 7, 12, and 16, indicating the influence of natural selection on environmental adaptation, disease resistance, and reproductive performance in the indigenous breed. The shared selection signal on BTA 12 between Nguni and Bonsmara is especially noticeable, and it could reflect either shared ancestral selection driven by similar environmental or production conditions. Similar findings were reported by [29], who identified a common region on BTA 12 and revealed the highest iHS score of 6.047 for Nguni and Bonsmara. Previous studies have shown that the number and strength of selection signatures vary among breeds. These differences are often linked to factors including breeding history, effective population size, and the intensity of artificial selection [30,31]. These shared genomic regions under selection may reflect convergent selection pressures arising from similar production environments, overlapping breeding objectives, or historical gene flow between geographically proximate populations [32,33]. The consistency of BTA 12 as a selection hotspot across independent studies strengthens the biological relevance of this region and suggests its involvement in traits of broad adaptive and economic importance [29]. Simmental cattle exhibited multiple iHS signals across several chromosomes, consistent with their long history of structured artificial selection for growth and carcass traits. Commercial *Bos taurus* breeds subjected to intensive directional selection frequently display widespread haplotype-based selection signals, reflecting ongoing fixation of favourable alleles [30,34]. This is supported by a study by [35] in Chinese Simmental beef cattle, which identified SNPs significantly associated with carcass weight and bone weight, which may influence growth and carcass traits. In contrast, Angus cattle exhibited only one significant region on BTA 18, with selection signals that were not as strong. This could be because desirable alleles were fixed in the past due to long-term selection. As a result, the iHS method might not have detected current positive selection. This was also supported by a study [36] using the iHS method in Hanwoo and Angus cattle using whole-genome sequence data, which reported 33 genomic regions for Angus as compared to 298 in Hanwoo cattle. However, across all breeds, twelve main genomic regions under selection were identified, illustrating the variety of selection pressures occurring within beef cattle populations. The identification of both breed-specific and shared selection signals emphasises the relationship between adaptation, management strategies, and specialised breeding strategies.

### 4.2. Between-Population Differentiation and Divergent Selection

Fst analysis revealed extensive genomic differentiation between Bonsmara and Nguni, as well as between Simmental and Angus, indicating divergent selection pressures among these breed pairs. A threshold of ~0.4 identifies SNPs that are moderately divergent. This is consistent with the breeds’ shared ancestry: Bonsmara was established by crossing Nguni with other *Bos taurus* lines; therefore, many genomic regions preserve similar allele frequencies. Furthermore, several candidate genes were associated with reproductive efficiency and disease susceptibility. These findings align with previous studies showing that indigenous and locally adapted cattle breeds often harbour selection signatures related to fertility, immune response, and resilience to environmental stressors [28,29]. This may be related to the fact that South African cattle are raised in areas that frequently experience drought, have dry seasons, lack adequate nutrition, and are vulnerable to a range of internal and external parasites as well as stock diseases [37]. For Simmental vs. Angus, a higher and more stringent cut-off of ~0.7 was observed, reflecting a substantially higher baseline differentiation. High Fst values are a sign of population-specific adaptation or divergent artificial selection, particularly when observed consistently across multiple genomic regions [38]. The genome-wide nature of differentiation observed in this study suggests that both environmental adaptation and breed-specific management practices have contributed to shaping distinct genetic architectures.

Gene annotation analyses using the iHS method highlighted the association of the FAM110B gene with QTLs for carcass weight and body conformation score in Simmental cattle. Ref. [39] reported the same gene on BTA 14 for carcass weight in Korean Hanwoo cattle. The gene FAM110B has been previously shown to affect several traits, such as growth, birth weight, average daily gain, feed intake, meat tenderness, height, stature, and carcass weight, in different beef cattle breeds [40]. The KCNQ3 gene was identified in association with QTLs related to important traits such as reproduction and disease resistance in Bonsmara vs. Nguni. The gene was also reported to be associated with milk, reproduction, and production traits in Nellore cattle [41]. The PAPPA gene was associated with sperm count and insemination per conception in Bonsmara vs. Nguni. The PAPPA gene was also reported on BTA 8 for Holstein-Friesian bulls, which was significant for several spermatozoa traits [42]. Mota et al. [37] reported that the PAPPA gene is strongly associated with female fertility. Another gene, GRIK2, has been reported to be related to the nervous system in Bashan cattle [43], as well as to the development of the nervous system in swamp buffalo, and has been reported to be related to reproductive functions through its effect on gonadotropin-releasing hormone (GnRH) secretion control [43]. The gene CHD7 was associated with the body conformation score in Bonsmara vs. Nguni. Ref. [44] reported that the CHD7 gene was detected at a 5% chromosome-wise level for carcass traits in Korean native cattle. This study also explored gene functions, revealing associations with cellular function, development, and reproduction. Some genes exhibited multiple functions, emphasising their multifaceted roles in various biological processes. The genes VASH2 and FOXO3 were identified in association with reproductive processes; this was supported by [45], which reported that VASH2 showed distinctive localisation and opposing functions on the fetoplacental vascularisation and regulation of cell fusion for syncytiotrophoblast formation. Similarly, FOXO3 has recently been reported to be essential in female fertility [46].

Functional enrichment analyses revealed that many candidate genes were involved in cellular organisation, developmental processes, reproduction, and homeostatic regulation. The overlap of genes across multiple biological processes indicates pleiotropic effects, whereby selection acting on one trait simultaneously influences several physiological systems. Such pleiotropy highlights the complex trait structure in livestock and emphasises the potential for trade-offs between productivity, adaptation, and resilience under selection.

## 5. Conclusions

In conclusion, this study identified selection signatures in South African Bonsmara and Nguni cattle breeds, as well as Simmental and Angus cattle breeds. This study further reported a common genomic region for selection signatures between Nguni and Bonsmara, providing insights into the genetic similarities and differences between the two breeds, which can be important for breeding programmes, genetic diversity studies, and understanding traits of interest. A shared selection signal on chromosome BTA 12 between Nguni and Bonsmara indicates a common genomic region potentially shaped by overlapping selective pressures. The study also adopted within- and between-population analyses to provide a comprehensive assessment of recent and historical selection processes. While previous studies have individually applied some of these approaches, this study uniquely combined them, providing a more holistic understanding of selection signatures and candidate genes in beef cattle breeds. The identified candidate genes associated with growth, reproduction, disease resistance, and physiological regulation underscore the relevance of these genomic regions for breeding and conservation strategies. Overall, these findings advance the understanding of selection dynamics and genetic differentiation in South African beef cattle and provide a foundation for genomic-assisted improvement programmes.

## Figures and Tables

**Table 1:** Integrated haplotype score (iHS) reveals within-population genomic regions under selection in beef cattle.

Breed	CHR	Gene Symbol	QTL	Ensemble Gene ID	ENTREZ	Position (MBP)	Description
SIM	2	*RBMS1*	-	ENSBTAG00000005180	526135	35.855477	*RNA-binding motif single-stranded interacting protein 1*
	6	*SRD5A3*	-	ENSBTAG00000014913	535834	70.803119	*steroid 5 alpha-reductase 3*
	6	*TMEM165*	-	ENSBTAG00000001269	532600	70.838726	*transmembrane protein 165*
	14	*FAM110B*	CW and BC	ENSBTAG00000050550	767946	24.365744	*family with sequence similarity 110 member B*
	17	*TBX5*	-	ENSBTAG00000011384	532970	60.228521	*T-box transcription factor 5*
BON	12	*CDK8*	-	ENSBTAG00000016737	507149	33.082022	*Cyclin-dependent kinase 8*
		*FLTI*	-	ENSBTAG00000016915	503620	31.624125	*Fms-related receptor tyrosine kinase 1*
NGU	12	*CDK8*	-	ENSBTAG00000016737	507149	33.082022	*cyclin-dependent kinase 8*
	12	*ENSBTAG00000019340*	-	ENSBTAG00000019340	NA	NA	*NA*
	12	*UBL3*	-	ENSBTAG00000012170	526950	30.806229	*ubiquitin-like 3*
	16	*PLA2G4A*	-	ENSBTAG00000013298	525072	67.906979	*phospholipase A2 group*
	16	*CRB1*	-	ENSBTAG00000008944	520406	76.188124	*crumbs cell polarity complex component 1*
	16	*VASH2*	-	ENSBTAG00000003701		70.601757	*vasohibin 2*
NGU&BON	12	*CDK8*	-	ENSBTAG00000016737	507149	33.082022	*cyclin-dependent kinase 8*

**Table 2:** Genomic differentiation and population-specific selection revealed by Fst analysis.

Breed	CHR	Gene Symbol	QTL	Ensemble Gene ID	ENTREZ	Position (MBP)	Description
NGU&BON	8	*PAPPA*	Sperm count and Insemination per conception	ENSBTAG00000004010	282647	105.3519	*pappalysin 1*
	9	*AFG1L*	-	ENSBTAG00000014592	537689	41.65511	*AFG1-like ATPase*
	9	*FOXO3*	-	ENSBTAG00000011234	535530	41.522588	*forkhead box O3*
	9	*GRIK2*	Bovine tuberculosis susceptibility and insemination per conception	ENSBTAG00000033153	615226	47.889245	*glutamate ionotropic receptor kainate type subunit 2*
	13	*PLXDC2*	-	ENSBTAG00000009475	515731	21.312388	*plexin domain containing 2*
	14	*ZNF704*	-	ENSBTAG00000021743	513243	43.837811	*zinc finger protein 704*
	14	*KCNQ3*	Bovine respiratory disease susceptibility calving ease and insemination per conception	ENSBTAG00000020667	281884	8.738181	*potassium voltage-gated channel subfamily Q member 3*
	20	*PLCXD3*	-	ENSBTAG00000010822	781239	781239	*phosphatidylinositol specific phospholipase C X domain-containing 3*
	22	*TCAIM*	-	ENSBTAG00000016622	515010	16.271889	*T cell activation inhibitor, mitochondrial*
	28	*FAM149B1*	-	ENSBTAG00000019130	533952	29.237038	*family with sequence similarity 149 member B1*
SIM vs. ANG	7	*FAM172A*	-	ENSBTAG00000050195	617002	93.088059	*family with sequence similarity 172 member A*
	10	*KCNK13*	-	ENSBTAG00000045849	787307	101.723038	*potassium two pore domain channel subfamily K member 13*
	13	*CHMP4B*	-	ENSBTAG00000013387	616164	63.226304	*charged multivesicular body protein 4B*
	19	*MYO1D*	-	ENSBTAG00000015527	522967	17.330801	*myosin ID*
	21	*MIPOL1*	-	ENSBTAG00000000655	528380	47.398496	*mirror-image polydactyly 1*

## Data Availability

The data supporting the findings of this study are available from DRYAD under the following DOI: https://doi.org/10.5061/dryad.qjq2bvqq3.
